# Bibliometric analysis of global research trends in magnetic resonance imaging studies of substantia nigra in Parkinson’s disease (2001–2024)

**DOI:** 10.3389/fnagi.2024.1455562

**Published:** 2024-09-03

**Authors:** Mei Jiang, Xu Deng, Zixiong Qiu, Jie Li, Zifan Song, Xiaoshuai Chen, Ruiqi Chen, Xianzhi Huang, Xiaojun Cui, Yuan Fu

**Affiliations:** ^1^Dongguan Key Laboratory of Stem Cell and Regenerative Tissue Engineering, Department of Human Anatomy, Dongguan Campus, Guangdong Medical University, Dongguan, China; ^2^School of Sports Health, Guangdong Vocational Institute of Sport, Guangzhou, China

**Keywords:** Parkinson’s disease, substantia nigra, magnetic resonance imaging, bibliometric analysis, CiteSpace, VOSviewer

## Abstract

**Background:**

Parkinson’s disease (PD) is a globally prevalent neurodegenerative disorder, primarily characterized by muscle rigidity, resting tremor, and bradykinesia. The incidence of PD is rapidly escalating worldwide. Numerous studies have been conducted on the application of magnetic resonance imaging (MRI) in investigating the substantia nigra (SN) in PD patients. However, to date, no bibliometric analysis has been performed on this specific research area. Therefore, this study aimed to provide a comprehensive analysis of the current status in MRI research on the SN in PD patients.

**Materials and methods:**

MRI study records related to the SN in PD patients from 2001 to 2024 were searched by using the Web of Science Core Collection (WOSCC) database and then the CiteSpace and VOSviewer were used to conduct bibliometric analysis.

**Results:**

Our analysis found that the number of published articles related studies on MRI of the SN in PD showed an overall upward trend over the past decade, in which Lehericy, Stephane, Du, Guangwei, and Huang, Xuemei are the top three authors with the most articles. Additionally, United States, China and Germany are the main contributors to MRI studies of SN in PD. And Shanghai Jiao Tong University, University of Florida and Seoul National University are the leading institutions in the field. Finally, the keyword analysis showed that the hotspots and trends of research in this field are mainly concentrated in quantitative susceptibility mapping, neuroimaging, and neuromelanin-sensitive MRI.

**Conclusion:**

These analysis identified the most influential authors, institutions, countries and research hotspots in the field of SN-MRI research in PD, which has reference significance for the research interest in this field and provides a new idea for PD prevention.

## 1 Introduction

Parkinson’s disease (PD), a widespread neurodegenerative disorder, is marked by progressive and debilitating alterations in the brain, exerting a significant impact on families and society (Chu et al., 2024; Costa et al., 2023). The primary pathological changes associated with PD involve the degeneration of dopaminergic neurons, especially in the substantia nigra (SN), a region of the brain integral to dopamine production (Arotcarena et al., 2022). These neuronal losses result in symptoms such as static tremors, muscle stiffness, bradykinesia, and postural gait disturbances, substantially affecting an individual’s daily activities and life quality (Morris et al., 2024; Leite Silva et al., 2023).

The advent of neuroimaging has seen magnetic resonance imaging (MRI) emerge as a fundamental tool for PD diagnosis (Martinez et al., 2023; Bae et al., 2021). MRI is effective in revealing atrophy in specific brain regions and alterations in the basal ganglia, which enhance the diagnostic accuracy of PD (Tuite, 2016; Hopes et al., 2016). Furthermore, advanced MRI techniques such as diffusion tensor imaging (DTI) can elucidate changes in white matter integrity (Modrego et al., 2011), while resting-state functional MRI (rs-fMRI) is utilized to assess functional connectivity in the brain, revealing network alterations in patients with PD (Campbell et al., 2015). The integration of these technologies provides a more comprehensive understanding of the pathological mechanisms underlying PD. Despite a growing awareness of the application of MRI in PD, there remains a lack of comprehensive understanding regarding the current research landscape, key areas of interest, and emerging trends within this field.

In recent years, the field of bibliometric analysis has witnessed widespread adoption in the study of academic publications (Hassan and Duarte, 2024). This method leverages the application of advanced mathematical and statistical techniques, as well as sophisticated bibliometric and data mining algorithms, to meticulously visualize the intricate co-citation network of scientific research (Chu et al., 2024). Through this multifaceted approach, researchers are empowered to not merely discern the current state of a given field, but also elucidate the emergent hotspots and trajectories of cutting-edge trends (Ninkov et al., 2022). In order to garner a more nuanced understanding of the evolving landscape surrounding the MRI of the SN in PD patients, we have undertaken a comprehensive bibliometric analysis of the relevant literature indexed in the Web of Science Core Collection (WOSCC) between 2001 and 2024.

## 2 Methods

### 2.1 Study design

The current study utilized the extensive WOSCC database to conduct a thorough search of relevant scholarly literature. Subsequently, the analytical tools VOSviewer (version 1.6.20) and CiteSpace (version 6.3) were employed for a rigorous bibliometric analysis. This multifaceted approach revealed a range of insightful trends, including the accumulated volume of published literature, the geographic distribution of contributing countries and institutions, the group of prominent authors, and the interconnected network of keyword associations.

### 2.2 Data acquisition

In this scholarly endeavor, the esteemed WOSCC database was meticulously queried to identify articles and reviews pertaining to the intersection of “Parkinson’s disease,” “substantia nigra,” and “MRI.” The search was conducted in the English language, with a specific cut-off date for the search set for the 6th of May, 2024. During the data processing phase, a curated selection of 750 scholarly works was meticulously screened, with the chosen content designated for download in its entirety, including all records and references, in a plain text format. These documents were sequentially labeled as “download_N,” with “N” representing a natural numeral. Upon their incorporation into the CiteSpace software for the purpose of deduplication, a total of 750 authentic pieces of literature were deemed eligible for inclusion. The temporal scope of this literature spans from the 1st of January, 2001, to the 6th of May, 2024, encompassing a rich tapestry of knowledge within the specified domain.

### 2.3 Statistical analysis

In this pursuit of academic refinement, the data was meticulously imported into the software of Excel, CiteSpace, and VOSviewer for a comprehensive visual analysis. The analytical parameters were meticulously calibrated with precision: the temporal division spanned from 2001 to 2024, with each year demarcated as a distinct unit for an annual examination. The types of nodes selected for this scholarly inquiry encompassed a diverse array, including authors, institutions, countries, and keywords, each contributing to a multifaceted exploration of the subject matter.

#### 2.3.1 Analysis of annual publication volume

First, we imported the data into CiteSpace software to remove duplicates. Then, we tallied the number of published papers for each year from 2001 to 2024 and created bar charts to compare the number of published papers in different years.

#### 2.3.2 Collaborative network analysis of countries, institutions, and authors

In VOSviewer, select the node type as Author, Country, or institutions and then draw the corresponding map. At the same time, the study also generates a national network map illustrating the number of publications.

#### 2.3.3 Analysis of journals and co-cited journals

Utilize the VOSviewer software to delineate a map by selecting “Sources” as the node type. The magnitude of the nodes within the graphical representation is indicative of the journal’s vibrancy and prominence within its academic sphere; an expanded node signifies greater activity. Furthermore, the interlinking lines illustrate the interconnections and cross-referencing dynamics among these scholarly entities.

#### 2.3.4 Keyword analysis

In VOSviewer software, the node type is “All keywords,” the frequency of keywords reflects the hotspot of the research field, and the size of the node is directly proportional to the frequency of occurrence. The larger the node, the higher the frequency of occurrence.

#### 2.3.5 Keywords co-occurrence time zone mapping analysis

In CiteSpace software, we imported the de-emphasized data to make the keyword co-occurrence time zone mapping. The time zone graph node size indicates the frequency and importance of the keyword in the field.

#### 2.3.6 Keyword co-occurrence timeline analysis

In CiteSpace software, we imported the de-emphasized data to make the keyword co-occurrence timeline mapping. Keywords from the same cluster on the timeline axis are placed on the same horizontal axis, and the node size indicates the frequency of occurrence as well as the importance of the clustered topic in this domain.

#### 2.3.7 Keyword emergence analysis

On the basis of keyword co-occurrence, the emergence analysis is carried out. The emergence analysis can show the keywords with a sudden increase in frequency in a short period of time, indicating the importance of the keywords in the field of research and the degree of attention, reflecting the research frontier and hotspots in a certain period of time. The longer the length of the emergence, the longer the duration of the keyword heat and the higher the attention.

## 3 Results

### 3.1 Exponential growth in SN-MRI research literature on PD

**Figure 1:**
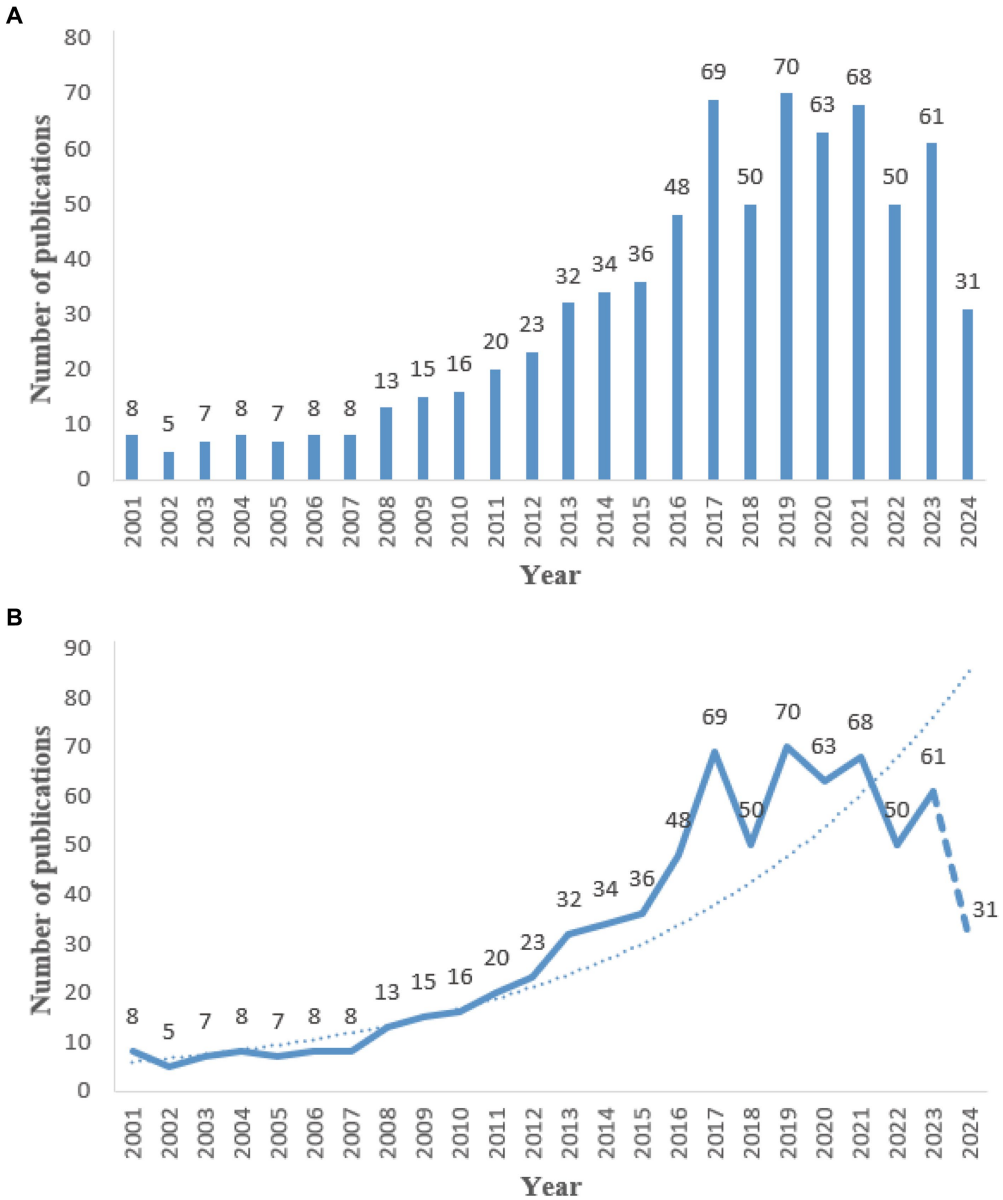
Annual number of publications from 2001 to 2022. **(A)** Bar chart of the number of publications per year. **(B)** Line chart of the number of publications per year.

The extant research literature on SN-MRI in PD patients has witnessed an exponential proliferation in recent years, the fitting curve described by the equation *y* = 4E-101e^0.1165x^. From the period spanning 2001 to 2010, the number of published works demonstrated an incremental trend, yet remained constrained below 20 individual contributions, amounting to an average of 9.5 publications *per annum*. However, the subsequent years, encompassing 2011 to 2023, have borne witness to a marked acceleration in the publication rate, with over 20 papers being disseminated annually. The total number of published works per year has now reached 48, reflecting a remarkable 505% increase during this interval. It is noteworthy that, given the cutoff date of the present search on May 6, 2024, only 31 published literatures are presently available for the year in question. Nonetheless, extrapolating from the established growth trajectory in this field, one may anticipate a continued escalation in the volume of publications to be expected in the future (see Figure 1).

### 3.2 Network analysis of the collaborative authors, institutions, and countries in SN-MRI research of PD

**Figure 2:**
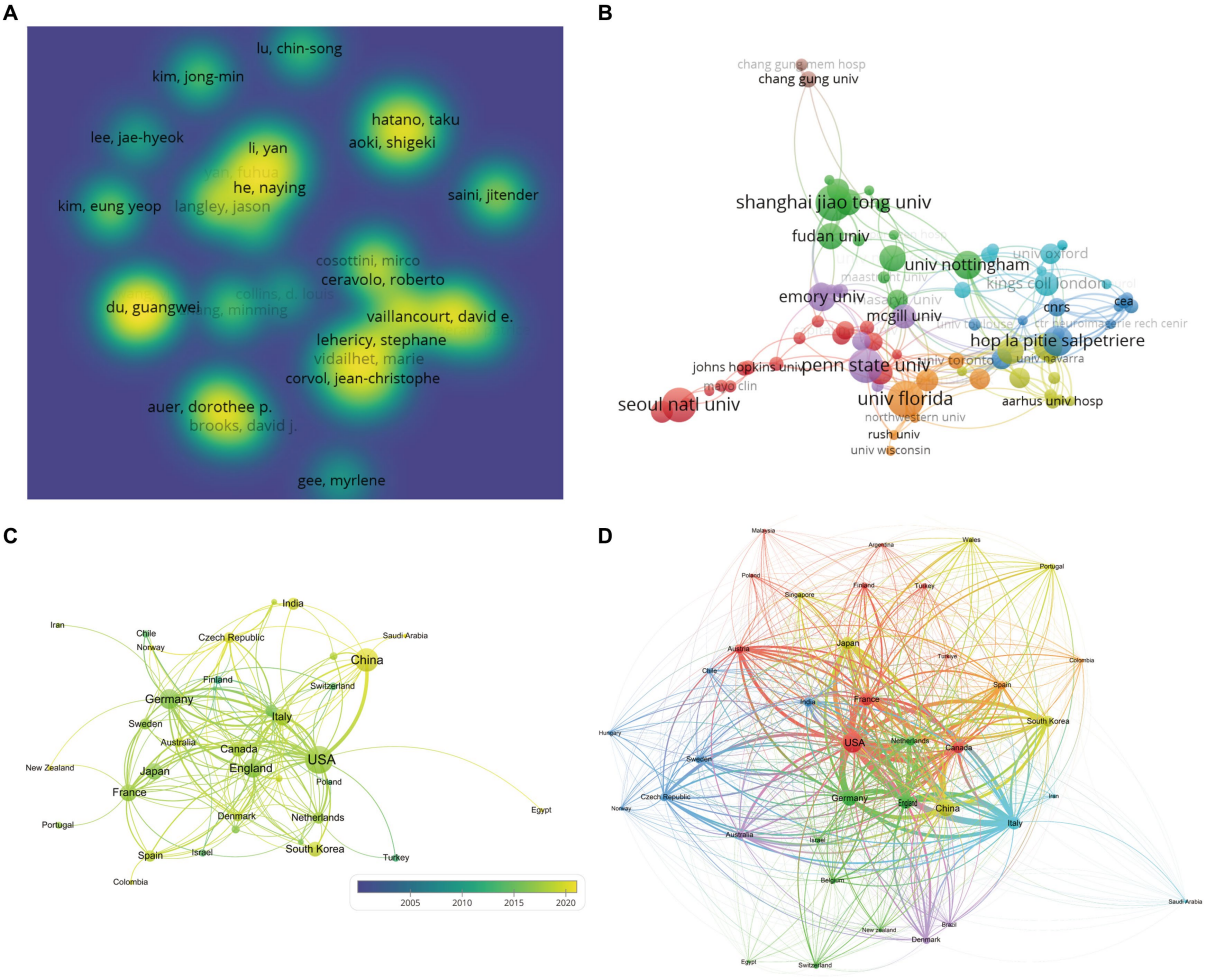
Map of the network of authors, institutions and countries. **(A)** Density view of the authors. **(B)** Clustering view of institutions. **(C)** Country cooperation network map. **(D)** Countries experiencing the most rapid growth in publication output.

The network diagram depicting the author collaborations in SN-MRI studies of PD patients revealed a robust network comprising 77 nodes and 219 connections (Figure 2A). The high density of nodes underscores the vibrant research activity in this field. The size of each node in the institutional network graph corresponds to the level of engagement and visibility of the respective institution. The larger the node, the greater the activity and prominence. Notably, Shanghai Jiao Tong University emerges as a hub of heightened research involvement in this domain (Figure 2B). Similarly, the national network map illustrates that the size of each node reflects the degree of activity and visibility, with the coloring denoting the temporal dimension and the connecting lines representing cross-references and collaborations between the nodes. As evidenced in Figure 2C, China has become increasingly engaged in this research area in recent years. Complementing the network analysis, Tables 1–3 succinctly summarizes the top 5 contributors in terms of publication output, encompassing the leading authors, institutions, and countries within this research field. Furthermore, the study illustrates the countries experiencing the most rapid growth in publication production (Figure 2D), while Table 4 makes a list of the top 5 countries experiencing the most rapid growth in publication output.

**Table 1:** Top 5 authors in terms of publications in the collaborative network.

Ranking	Author	Documents	Citations	Total link strength
1	Lehericy, Stephane	19	1,059	85
2	Du, Guangwei	16	587	51
3	Huang, Xuemei	16	587	51
4	Lewis, Mechelle m	16	587	51
5	Yan, Fuhua	15	470	77

**Table 2:** Top 5 institutions in terms of publications in the collaborative network.

Ranking	Institution	Documents	Citations	Total link strength
1	Shanghai Jiao Tong Univ	18	532	22
2	Univ Florida	18	890	18
3	Seoul Natl Univ	17	437	10
4	Penn State Univ	17	661	2
5	Hop La Pitie Salpetriere	15	1,026	31

**Table 3:** Top 5 countries in terms of publications in the collaborative network.

Ranking	Country	Documents	Citations	Total link strength
1	USA	211	9,537	151
2	China	122	2,403	49
3	Germany	91	3,810	93
4	England	82	4,918	90
5	Italy	60	3,311	74

**Table 4:** Top 5 countries experiencing the most rapid growth in publication output.

Ranking	Country	Documents	Citations	Total link strength
1	USA	211	9,537	181,523
2	China	122	2,403	97,619
3	Germany	91	3,810	88,020
4	England	82	4,918	92,524
5	France	60	3,862	79,695

### 3.3 Analysis of journal publication output and citation in the field of SN-MRI in PD

**Figure 3:**
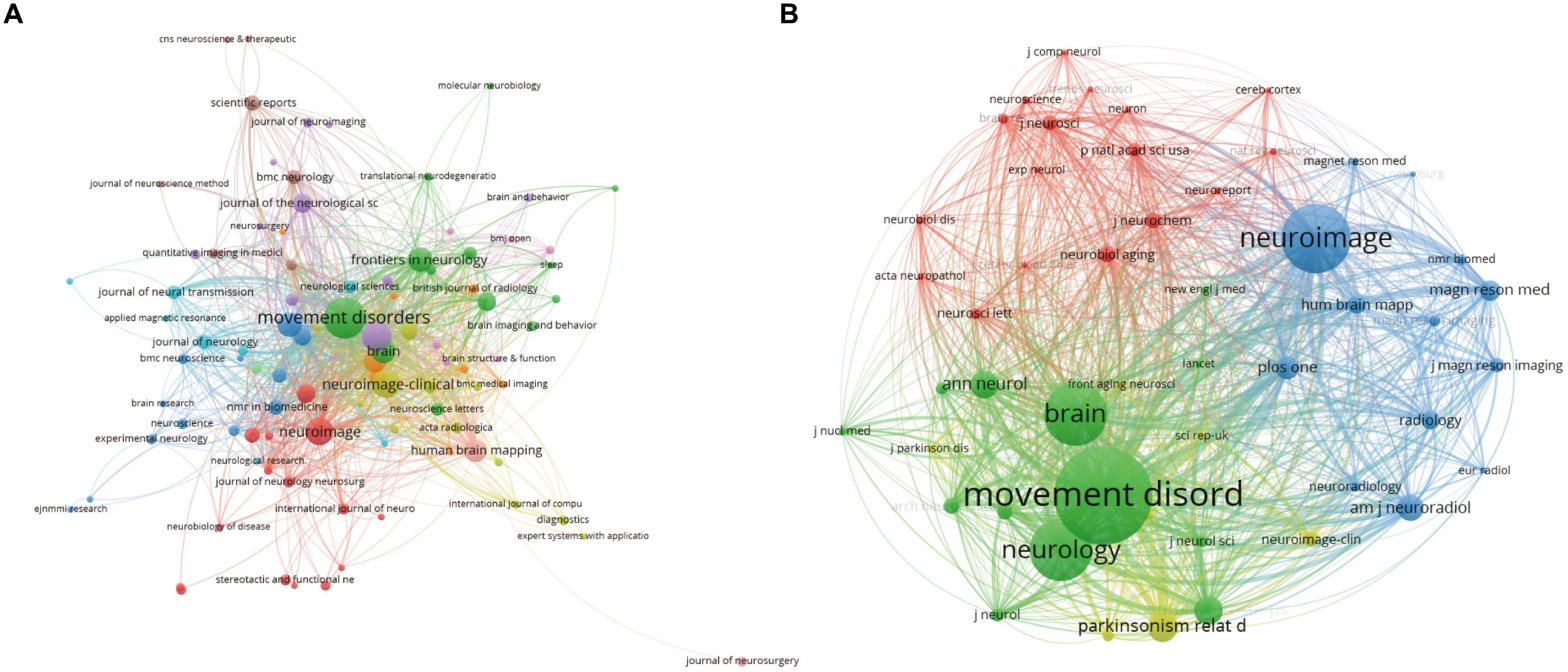
Network map of co-cited journals. **(A)** Network map of journals with the highest number of relevant literature. **(B)** Network map of journals with the highest number of cited literature.

**Figure 4:**
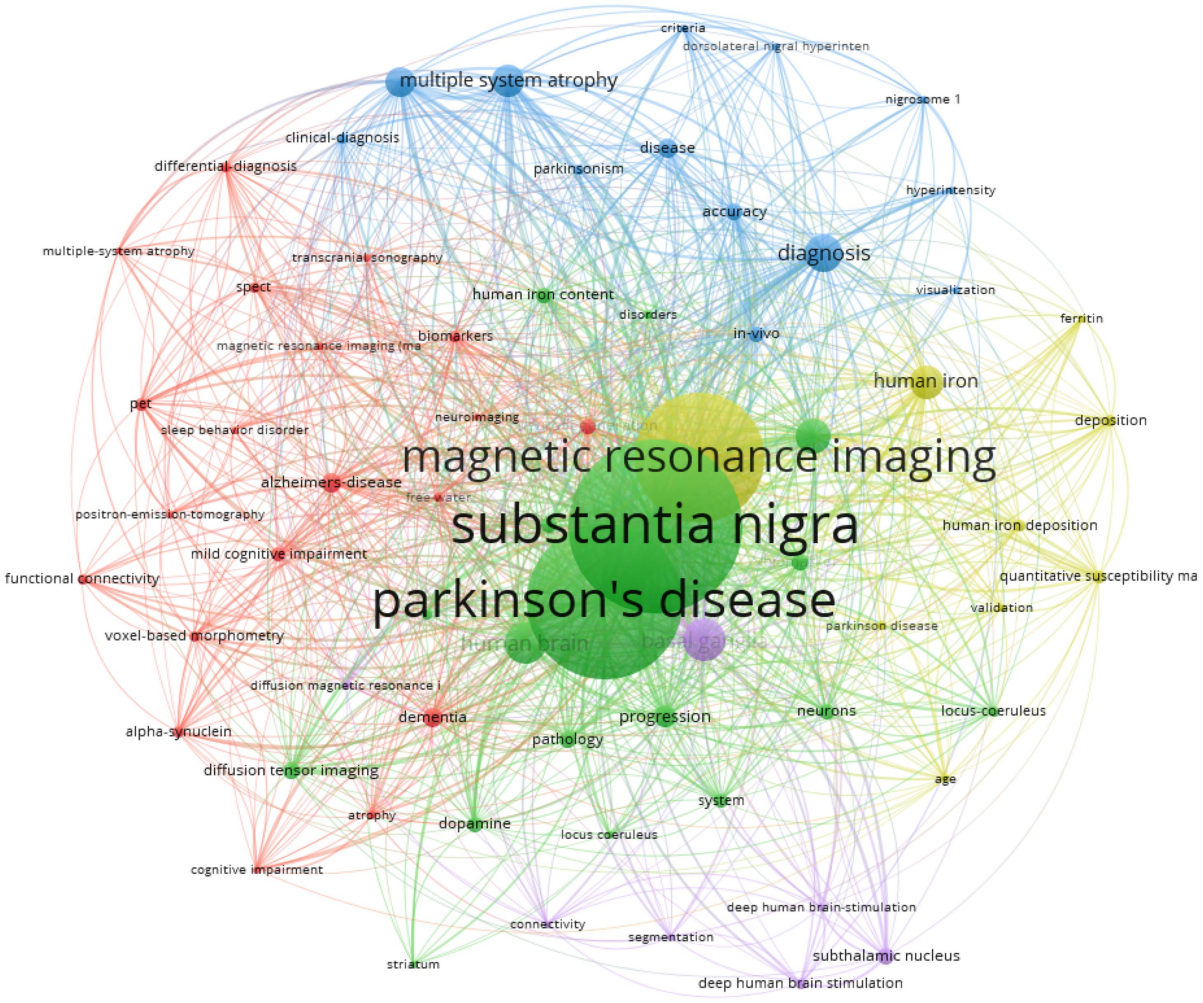
Keyword co-occurrence network diagram. The larger the circle shows the more prominent the keyword is in the network graph. The colours represent clustering.

The collaborative network of journals depicted in VOSviewer is showcased in Figure 3. As illustrated in Table 5, the five journals boasting the most prolific publication output are as follows: *Movement Disorders*, *Parkinsonism & Related Disorders*, *NeuroImage—Clinical*, *Brain*, and *Frontiers in Neurology*. The impressive lead held by *Movement Disorders* in this regard is immediately evident. The co-citation network of journals, presented in Figure 4, elegantly resolves into four distinct clusters, each denoted by a unique color. Of the 3,005 journals surveyed, a total of 50 garnered over 160 citations. As summarized in Table 6, the five most heavily cited journals are *Movement Disorders*, *NeuroImage, Brain*, *Neurology*, and *Parkinsonism & Related Disorders*.

**Table 5:** The top 5 journals with the highest number of articles in the journals.

Ranking	Journals	Documents	Citations	Total link strength
1	Movement disorders	65	3,626	1,108
2	Parkinsonism and related disorders	32	806	381
3	NeuroImage—Clinical	30	932	491
4	Brain	29	1,503	429
5	Frontiers in neurology	23	1977	430

**Table 6:** Top 5 most co-cited journals.

Ranking	Jounals	Citations	Total link strength
1	Movement disorders	3,588	183,582
2	NeuroImage	2,483	120,395
3	Brain	2,235	124,779
4	Neurology	2,209	118,309
5	Parkinsonism and related disorders	1,019	65,342

### 3.4 Keyword co-occurrence analysis of SN-MRI in PD

Concise keywords encapsulate the crux of a scholarly work, and meticulous keyword co-occurrence analysis can elucidate the prevailing research foci within a given field. The size of each circular node corresponds to the relative frequency of the associated keyword, underscoring its prominence and representative significance. The interconnecting lines between nodes denote the strength of the conceptual linkages, whereby thicker lines indicate a more robust co-occurrence rate across the corpus. The distinct node colorations demarcate the emergent thematic clusters, illuminating the multifarious research trajectories. This keyword co-occurrence network graph catalogues only those lexical units exceeding 20 instances, comprising a total of 1,682 connective strands. The keyword commanding the highest frequency of appearance was “substantia nigra,” closely followed by “Parkinson’s disease,” “magnetic resonance imaging,” “basal ganglia,” “human brain,” “diagnosis,” “neuromelanin,” “human iron,” “multiple system atrophy,” and “progressive supranuclear palsy.” Detailed information can be found in Table 7.

**Table 7:** Top 10 keywords in terms of frequency.

Ranking	Keyword	Frequency	Total link strength
1	Substantia nigra	481	2,483
2	Parkinson’s disease	432	2,183
3	Magnetic resonance imaging	359	1957
4	Basal ganglia	120	627
5	Human brain	117	600
6	Diagnosis	106	628
7	Neuromelanin	99	641
8	Human iron	95	585
9	Multiple system atrophy	92	577
10	Progressive supranuclear palsy	84	541

### 3.5 Timezone analysis of keyword trends of SN-MRI in PD

**Figure 5:**
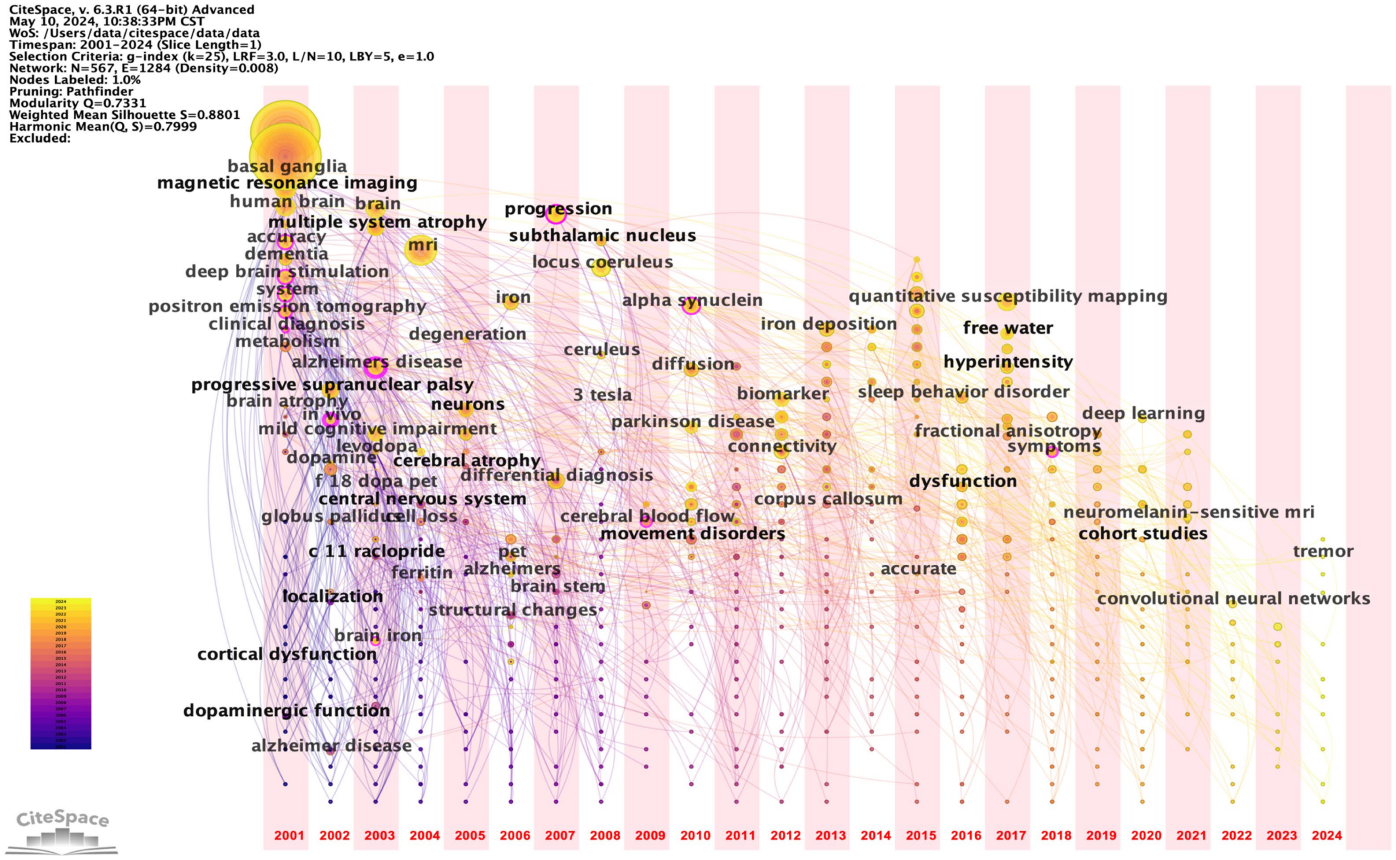
Keywords time zone map. The larger the circle, the more prominent the node is in the network graph; the colour represents the year.

The keyword timezone analysis reveals that research on basal ganglia structures (including the SN), MRI, and PD has sustained a high level of interest over time. Conversely, the emergence of deep learning and neuromelanin-sensitive MRI in recent years may signify an emerging research direction within the field (see Figure 5).

### 3.6 Timeline analysis of keyword trends of SN-MRI in PD

**Figure 6:**
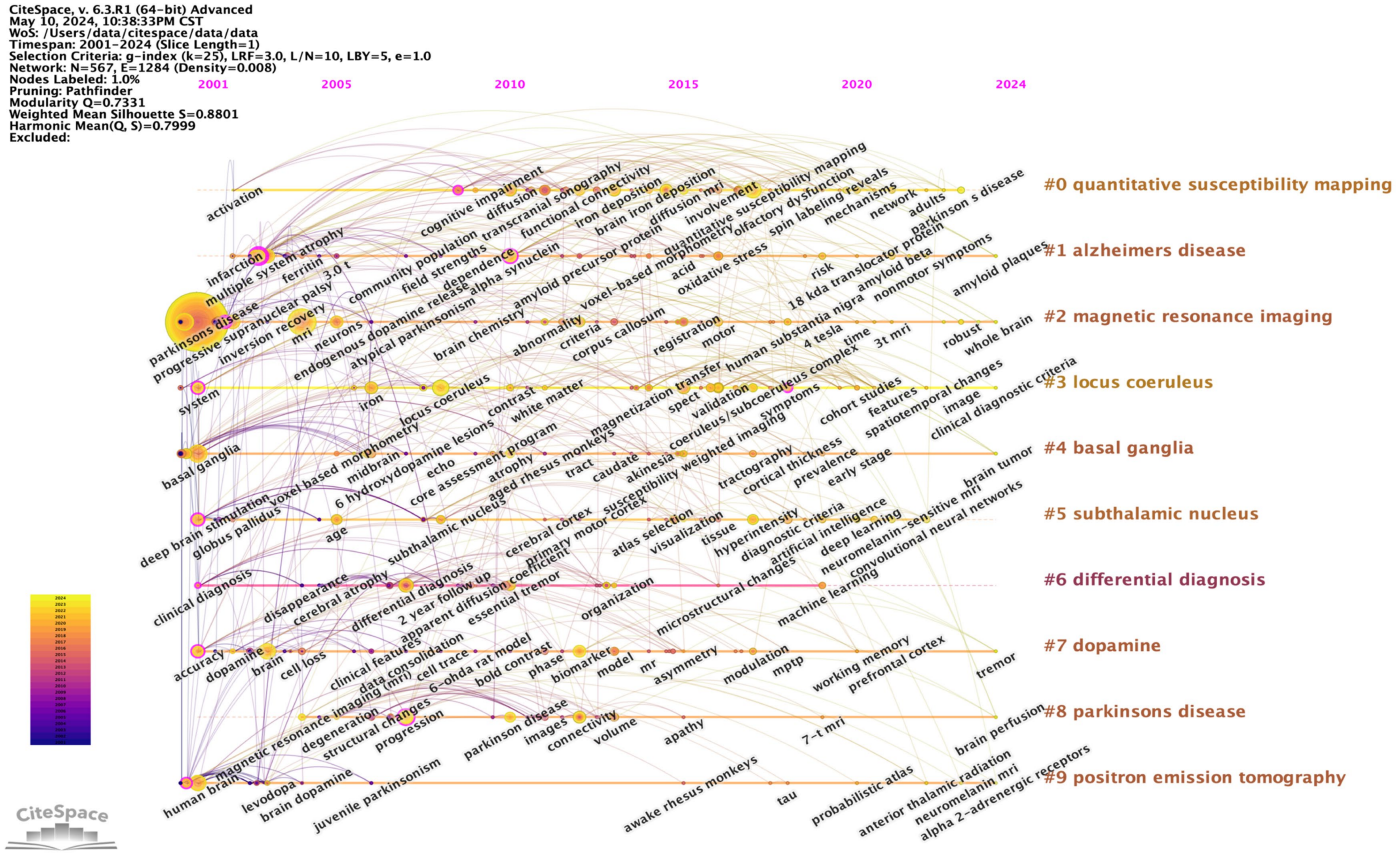
Keyword Timeline Chart. The larger the circle shows the more prominent the node is in the network graph and the colour represents the year.

The keyword timeline graph illuminates uninterrupted scholarly inquiry into PD, MRI, and allied domains. Within Cluster 2, the nodes denoting the keywords “Parkinson’s disease,” “MRI” and “substantia nigra” manifest enhanced prominence, signifying that research avenues encompassed by these terms have sustained a heightened level of momentum. Moreover, the predominance of yellow concentric circles intimates that these focal areas have witnessed heightened activity in recent years. Perusal of the keyword timeline mapping further elucidates that basal ganglia, dopamine, and quantitative susceptibility mapping have emerged as veritable hotbeds of research inquiry within this scholarly domain (see Figure 6).

### 3.7 Keywords with the strongest citation bursts of SN-MRI in PD

**Figure 7:**
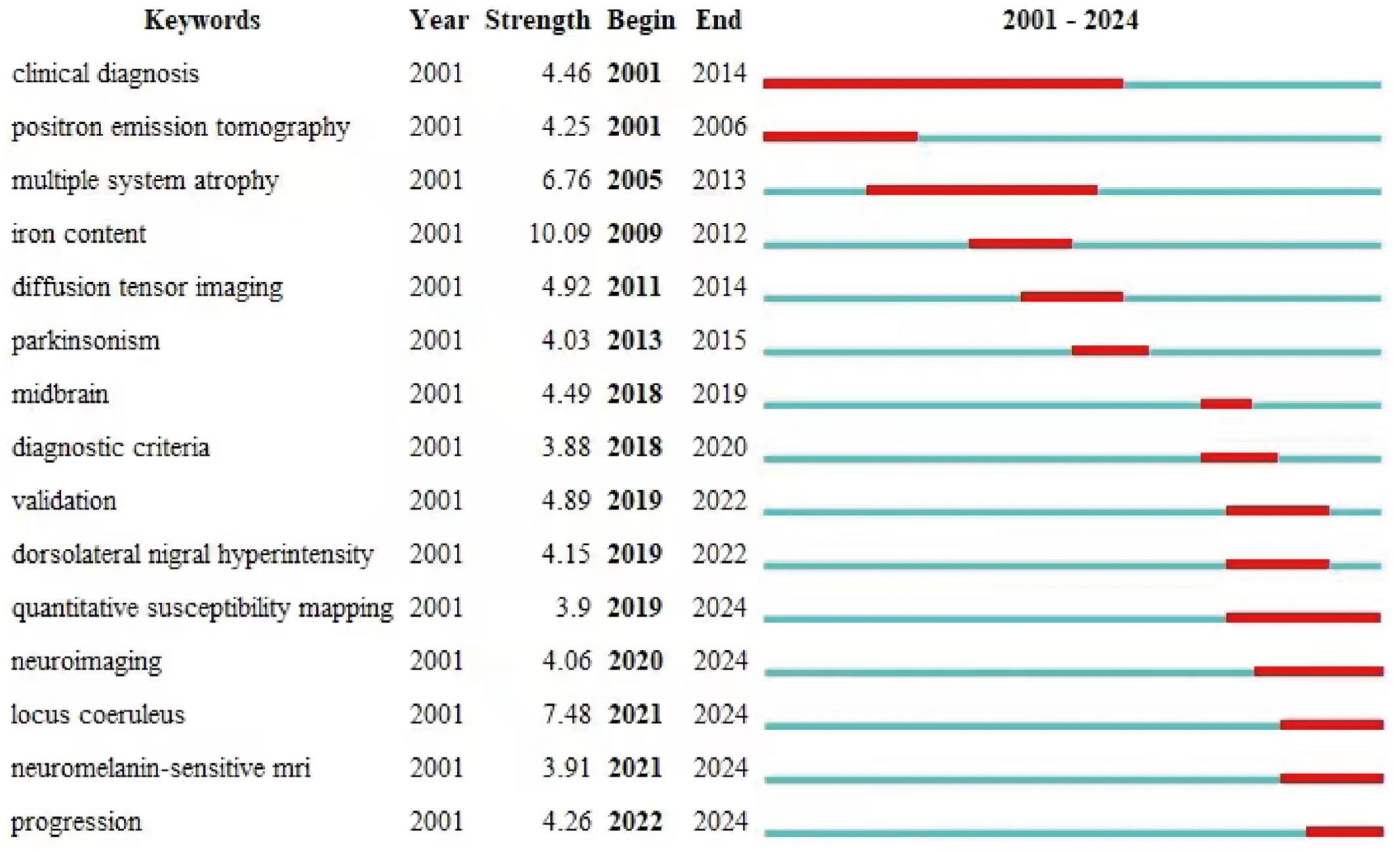
Top 15 Keywords with the strongest citation bursts. The length of the red line segment represents the duration of the outbreak for that keyword.

The top 15 keywords were meticulously selected and analyzed, and those that exhibited consistent, prolonged prominence throughout the 24-year period included clinical diagnosis, positron emission tomography, and multiple system atrophy, suggesting that these aspects of research have long been central focal points within the field. In the most recent 4-year interval, emerging hotspots have manifested, as evidenced by the keywords emphasizing neuroimaging, locus coeruleus (LC), neuromelanin-sensitive MRI, and progression, among others (Figure 7).

## 4 Discussion

### 4.1 Basic information

Based on the WOSCC database, this study employs bibliometric techniques to analyze existing research literature related to SN MRI assessment of PD from 2001 to 2024. Through this comprehensive analysis of publication volumes, authorship, institutional affiliations, geographic provenance, and keyword trends, this study aims to elucidate the current state of scholarship in this domain, identify nascent research foci, and provide a robust foundation to guide future inquiry.

Over the past 24 years, there has been a significant surge in the application of MRI to explore the SN in patients suffering from PD. The international cooperation network paints a vibrant picture of collaborative efforts, with China, the United States, the United Kingdom, and Germany emerging as key nodes. These countries have established extensive transnational collaborations, with the United States playing a crucial role as a bridge among various participating nations. This amalgamation of global expertise has undeniably augmented our comprehension of MRI-based studies of the SN in PD patients. Among the top five authors, Lehericy stands out with the highest publication record that have garnered 1,059 citations, which demonstrates his significant contributions to the fields of PD and MRI. Prof. Lehericy believes that various imaging techniques are applicable at different stages of PD. Specifically, diffusion imaging is instrumental for early diagnosis of PD, while free-water imaging serves as a valuable tool for tracking disease progression (Mitchell et al., 2021). Overall, SN-MRI is experiencing a rapid growth trend in PD research, in which exists a robust collaborative network among authors, institutions, and countries involved in this field.

### 4.2 Research frontiers and hotspots

In the realm of global research trends pertaining to MRI studies of the SN in PD patients, a comprehensive exploration can be achieved through an examination of keyword prominence, timelines, and other pertinent metrics. Our meticulous investigation has unveiled that state-of-the-art techniques such as Quantitative Susceptibility Mapping (QSM), advanced neuroimaging modalities, and the scrutiny of the LC alongside neuromelanin-sensitive MRI have surfaced as rapidly expanding areas of concentration in the current research panorama.

PD represents a prevalent and debilitating neurodegenerative disorder affecting the central nervous system (Morris et al., 2024; Mitchell et al., 2021). Through the application of advanced neuroimaging techniques, researchers have elucidated disease-specific structural and functional aberrations. A cardinal pathological hallmark of PD manifests in the deposition of iron within the SN (Sheth et al., 2019). Nigrostriatal vesicles exhibiting reduced iron content demonstrate heightened signal intensity on magnetic susceptibility-weighted imaging sequences, in distinct contrast to the muted signal of the surrounding stroma—a radiographic feature colloquially referred to as the “swallow-tail sign” (Chau et al., 2020). Intriguingly, the absence of this characteristic “swallow-tail sign” on 3.0 Tesla MRI has proven instrumental in discriminating healthy control subjects from those afflicted by PD (From the American Association of Neurological Surgeons (AANS) et al., 2018).

The emergence of QSM has facilitated the non-invasive *in vivo* quantification of cerebral iron content via MRI techniques (Guan et al., 2024; He et al., 2022). QSM enables the quantitative assessment of tissue magnetization properties, thereby enabling a more objective evaluation of iron deposition within the SN. Leveraging the quantitative analytical capabilities of QSM to assess SN lesions has been shown to enhance the sensitivity and specificity of PD diagnosis (Cheng et al., 2019; Marxreiter et al., 2023).

The neuromelanin (NM) is known to play a pivotal role in the pathogenesis of PD (Mitra et al., 2023). Intriguing studies have demonstrated that overexpression of the enzyme tyrosinase within the SN of rodents induces an age-dependent synthesis of humanlike NM within dopaminergic neurons. The subsequent accumulation of this humanlike NM above a critical threshold appears to be inextricably linked with the emergence of a PD-like phenotype, the formation of Lewy bodies, and eventual neurodegeneration (Pizarro-Galleguillos et al., 2022; Carballo-Carbajal et al., 2019). Notably, the unique magnetic properties of NM, including a T1 shortening effect and magnetization transfer phenomenon, enable the sensitive detection of NM-rich dopaminergic neurons within the SN using specialized MRI techniques (Lee et al., 2016).

The insidious pathological hallmarks of PD encompass the inexorable loss of dopaminergic neurons within the SN (Peng et al., 2022; Pandian et al., 2022). Moreover, the number of neurons in the LC has been significantly reduced in patients with this serious neurodegenerative disorder, with losses ranging from 21 to 94% (Sun et al., 2023; Oertel et al., 2019; Giguere et al., 2018). Notably, neuromelanin-sensitive MRI can visualize the LC adjacent to the floor of the fourth ventricle at the level of the pontine tegmentum. The voxel signal intensity is closely correlated with the number of neuromelanin-rich neurons that comprise the LC (Keren et al., 2015; Madelung et al., 2022).

The research landscape surrounding MRI of the SN in SN predominantly centers on elucidating disease mechanisms and investigating therapeutic avenues. This concise review synthesizes the prevailing research foci and trajectories within this domain, with the aim of furnishing valuable insights to scholars engaged in this burgeoning field of inquiry.

### 4.3 Advantage and limitation

This research endeavor boasts several noteworthy strengths. (1) It represents the inaugural bibliometric analysis focused on the burgeoning field of MRI investigations into the SN in PD. Through rigorous statistical scrutiny and meticulous survey of the relevant literature, the study illuminates the global standing and impending trajectories of this dynamic domain. (2) The elucidation of the key contributing authors, nations, and institutions furnishes invaluable guidance for scholars seeking to forge deeper collaborative ties and advance research in this area. (3) The synthesis of keyword analyses astutely distills the current hotbeds of inquiry and cutting-edge frontiers, offering feasible research avenues and promising clinical directions for both clinicians and scientists. These insights into the field’s prevailing themes and emergent trends hold profound implications for enhancing early detection, diagnosis, and therapeutic interventions for PD. By meticulously analyzing and eloquently expounding upon the keywords and leading-edge focal points, the authors elucidate their nuanced understanding of MRI-based SN investigations in PD, with the aspiration of furnishing valuable guidance for both clinical and scientific research endeavors.

Concomitantly, it is judicious to acknowledge the inherent constraints of this scholarly endeavor. (1) The corpus of articles incorporated within this study is exclusively sourced from the prestigious WOSCC database. Although prior research has compellingly demonstrated that bibliometric analyses predicated upon the WOSCC database are scientifically robust and practicable, it is salient to note that not all pertinent findings within the field may be fully encapsulated. It is important to acknowledge that there are various other public and commercial bibliometric databases. None of these databases can be deemed superior, as citation data can vary significantly across platforms (Hirsch, 2005; Falagas et al., 2008). (2) This investigation is confined to the examination of original articles and reviews published exclusively in the English language. (3) While the utilization of VOSviewer and CiteSpace furnishes an objective analysis, there remains the potential for residual subjective judgments attributable to the manual screening of extant literature. Moreover, citation counts serve as a key indicator of scholarly impact. Employing a strategy that focuses on the absolute number of citations for a given article may disproportionately benefit earlier publications. Additionally, factors such as self-citation by journals and authors, incomplete citations, and citation omission bias can significantly influence citation rates (Brandt et al., 2019).

## 5 Conclusion

This study leverages the VOSviewer and CiteSpace software to provide an in-depth, multi-faceted analysis of the substantive body of research pertaining to the application of MRI techniques in the investigation of the SN in PD over the past two decades. The work systematically examines the evolving trends, core authors, high-impact nations and institutions, seminal journals, and pivotal keywords defining this dynamic research domain. The analysis illuminates the field’s current preoccupations and areas of heightened focus, including QSM, advanced neuroimaging modalities, the LC, and neuromelanin-sensitive MRI.

## Data Availability

The original contributions presented in the study are included in the article/supplementary material, further inquiries can be directed to the corresponding authors.
